# Spray drying as a strategy for biosurfactant recovery, concentration and storage

**DOI:** 10.1186/2193-1801-3-49

**Published:** 2014-01-24

**Authors:** Gisely S Barcelos, Lívia C Dias, Péricles L Fernandes, Rita de Cássi R Fernandes, Arnaldo C Borges, Karlos HM Kalks, Marcos R Tótola

**Affiliations:** Departamento de Microbiologia, Universidade Federal de Viçosa. ECS, sala 141. Campus da UFV, Centro, Viçosa, MG 36570000 Brazil

**Keywords:** Biosurfactant, Spray drying, Maltodextrin, Clay, Environmental biotechnology, Surfactin

## Abstract

The objective of this study was to analyze the use of Spray Drying for concentration and preservation of biosurfactants produced by *Bacillus subtilis* LBBMA RI4914 isolated from a heavy oil reservoir. Kaolinite and maltodextrin 10DE or 20DE were tested as drying adjuvants. Surface activity of the biosurfactant was analyzed by preparing dilution x surface activity curves of crude biosurfactant, crude biosurfactant plus adjuvants and of the dried products, after their reconstitution in water. The shelf life of the dried products was also evaluated. Spray drying was effective in the recovery and concentration of biosurfactant, while keeping its surface activity. Drying adjuvants were required to obtain a solid product with the desired characteristics. These compounds did not interfere with tensoactive properties of the biosurfactant molecules. The dehydrated product maintained its surfactant properties during storage at room temperature during the evaluation period (120 days), with no detectable loss of activity.

## Introduction

Biosurfactants are surface active metabolites produced by various micro-organisms, which may either remain attached to the cell surface or be secreted into the culture medium. These molecules, like any surfactant, lower the surface tension of a liquid, the interfacial tension between two liquids, or that between a liquid and a solid, acting as detergents, wetting agents, emulsifiers, foaming agents, and dispersants. The surfactants of microbial origin are a diverse group of molecules and are known to occur in a wide variety of chemical structures, such as glucolipids, lipopeptides, lipoproteins, fatty acids, neutral lipids, phospholipids, and polymeric or particulate structures. The characteristics that make biosurfactants a promising alternative to synthetic surfactants are their low critical micelle concentration (CMC) (Ghojavand et al. 2008), low toxicity (Lima et al. 2011a), high biodegradability (Lima et al. 2010) and high stability under extreme conditions of pH, salinity and temperature (Desai & Banat 1997). Moreover, unlike the synthetic surfactants, which are usually obtained from petroleum, biosurfactants can be produced in microbial fermentation processes from renewable and low cost substrates (Fox & Bala 2000;Maneerat 2005;Mukherjee et al. 2006;Nitschke et al. 2005;Nitschke & Pastore 2006).

Given the aforementioned advantages, biosurfactants have the potential to be used in many fields, including agriculture and food industries, chemical and pharmaceutical industries, cosmetics, etc. (Banat et al. 2010;Muthusamy et al. 2008;Soberón-Chávez & Maier 2011). Furthermore, many of the properties of biosurfactants, as emulsification/demulsification, dispersion, foaming, wetting, etc., make them ideal for use in many environmental processes (Pacwa-Płociniczak et al. 2011). These include remediation of organic contaminants such as petroleum hydrocarbons (Lima et al. 2011b;Maneerat 2005) and pesticides (Wattanaphon et al. 2008), remediation of heavy metals-contaminated areas (Das et al. 2009;Lima et al. 2011b;Mulligan et al. 2001), enhanced oil recovery (Youssef et al. 2007), among others.

Despite having all the desirable characteristics of a surfactant, the biosurfactants are not widely commercialized. This is due mainly to the high costs of production and purification and of low yields (Mukherjee et al. 2006), which makes the price of the final product less competitive with synthetic surfactants. However, for many applications in environmental biotechnology, purification is not necessary, what reduces considerably the cost of production and enables the use of such compounds on a large scale. In this case, storage and transportation logistics of the product to the site where it must be used become the main obstacles, since without a prior concentration step, a large volume of liquid needs to be transported under controlled conditions.

The best option for transportation of any product is in solid state, which minimizes the volume, losses by thermal or microbial degradation, and eliminates the need for special storage conditions. In this work, we applied the technique of spray drying for the recovery and concentration of biosurfactants. This process, widely used in food and pharmaceutical industries, consists of three basic steps: (i) first, the fluid is dispersed as microdroplets, increasing the surface area, (ii) the droplets are placed in contact with a stream of heated air; the large surface area of the microdroplets propitiates an efficient heat transfer; (iii) in the final step, solvent evaporation occurs, resulting in the formation of solids containing the product of interest (Broadhead et al. 1992;Masters 1985;Nonhebel & Moss 1971;Rankell et al. 2001;Shaw 1997). This work is based on the assumption that this technique can not only solve the problem of transportation and storage logistics of biosurfactants, but also contribute to reduce the cost of their production and use.

## Materials and methods

### Bacterial isolate and growth conditions

The bacterium used in the experiments was *Bacillus subtilis* LBBMA RI4914 (GenBank accession no. KF945169), belonging to the culture collection of the Laboratory of Biotechnology and Biodiversity for the Environment (LBBMA), Department of Microbiology, Universidade Federal de Viçosa, Viçosa, Minas Gerais - Brazil. This bacterium was isolated from a heavy oil reservoir from Itaúnas’s Oilfield, Conceição da Barra - ES - Brazil. *B. subtilis* LBBMA RI4914 produces Surfactin, as confirmed by infrared spectrum and NMR analysis of the biosurfactant molecules (data not shown). The strain was maintained in glycerol-TSA at -80°C. The bacterium was revived on LB agar for 24 h at 30°C, transferred to LB broth and incubated for 24 h at 30°C and 200 rpm. Inoculum was produced by cultivating the bacterium twice in mineral medium M2 (g L^-1^: hydrolyzed casein, 10.0; KH_2_PO_4_, 1.5; Na_2_HPO_4_, 4.0; MgSO_4_, 0.2; CaCl_2_.H_2_O, 0.013; C_6_H_5+4y_Fe_x_N_y_O_7_, 0.005; glycerol, 38.0) for 18 h at 30°C and 200 rpm. For biosurfactant production, the inoculum was centrifuged, washed twice in saline (NaCl 8.5 g L^-1^) and then used to inoculate two liters of mineral medium M2. The culture was incubated for 48 h at 30°C and 200 rpm in an orbital shaker. The culture was autoclaved at 121°C for 20 min and the resulting crude extract was used as the surfactant solution in the following experiments.

### Spray drying

Spray drying of crude biosurfactant was done in a bench top Mini Spray Dryer Buchi model B-191. The equipment was configured with inlet temperature of 170°C and exhaust temperature of 65°C. Flow dynamics of the apparatus was adjusted to obtain a suction of 0.83% and a pumping of 0.25%.

#### Drying adjuvants

Given the low concentration of solids and high concentration of organic acids in the crude biosurfactant, and hence low glass transition temperature of the dry solid, it was necessary to use drying adjuvants to obtain a product with low moisture content. Tested adjuvants were kaolinite (100 or 200 g L^-1^), and maltodextrin with different DE (dextrose equivalents). Maltodextrin is a polysaccharide that is used as a food additive. It is produced from starch by partial hydrolysis. Maltodextrin consists of D-glucose units connected in chains of variable length. The glucose units are primarily linked with α(1 → 4) glycosidic bonds. Maltodextrin is typically composed of a mixture of chains that vary from three to seventeen glucose units long. Maltodextrin 10DE was tested at 250 g L^-1^ and maltodextrin 20DE at 100 or 250 g L^-1^. The effect of the drying adjuvant on the tensoactive properties of biosurfactant was evaluated by preparing a curve of surfactant dilution x surface tension (CMD curve, item 2.3) of the crude biosurfactant in presence of drying adjuvants at the initial concentrations previously reported. The data were compared with those obtained with the crude biosurfactant extract without the addition of the drying adjuvants.

### Surface activity and critical micellar dilution of crude biosurfactant

Surface activity and relative concentration of biosurfactant in crude extracts, exposed or not to spray drying, were evaluated by determining the Critical Micellar Dilution (CMD), the dilution above which the concentration of surfactant molecules becomes lower than the Critical Micellar Concentration (CMC). CMD was determined by measuring surface tension of increasing dilutions of the autoclaved crude biosurfactant. Surface tension (ST) was determined by the Wilhelmy plate method, using an automatic tensiometer (Dataphysics, model DCAT-11). The CMD was defined as the dilution above which surface tension starts to increase (Risch & Reuneccius 1993).

### Analysis of spray dried products

The dried products were diluted in distilled water in order to achieve the same concentration of biosurfactant and adjuvants contained in the crude extracts before drying. Thus, 1.0 g or 2.5 g of clay or maltodextrin-containing dried products (samples from treatments with 100 and 250 g L^-1^ of kaolinite and maltodextrin, respectively), were dissolved in 10.0 mL of distilled water. After solubilization, the samples were diluted for surface-tension and CMD determination (Section 2.3). The results were compared with those obtained with crude surfactant before spray drying. Analyses were made one day after spray drying.

To check the shelf life and maintenance of surface-active properties of spray dried biosurfactants, the products were stored in white plastic flasks for four months at room temperature and humidity. After this period, the products were reconstituted in distilled water (item 2.4), following ST and CMD analysis, as described in Section 2.3.

## Results and discussion

### Drying adjuvants

Drying of crude biosurfactant produced by *B. subtilis* LBBMA RI4914 (and possibly other biosurfactants) without adjuvants resulted in a viscous, pale yellow and difficult to manipulate product, which stuck on the walls of the equipment. According to Adhikari et al. (Adhikari et al. 2003), the formation of a product with such characteristics, during dehydration of microbial cultures, is attributed to the presence of products of microbial metabolism (organic acids, proteins and fatty acids) and to the low concentration of solids.

**Figure 1:**
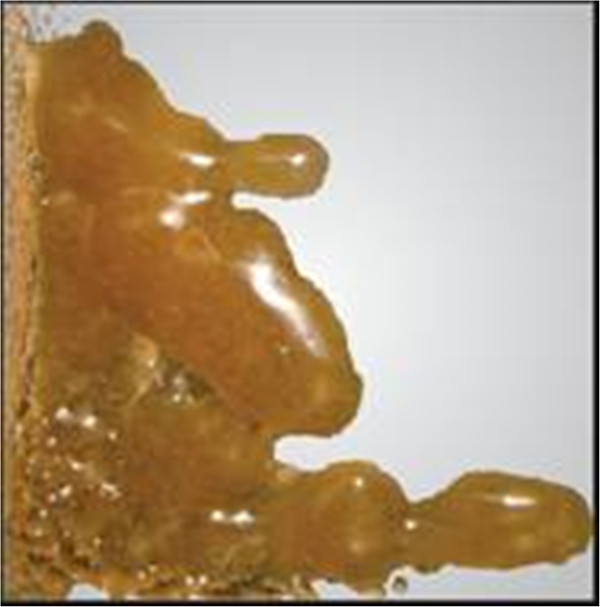
**Solid obtained after spray drying autoclaved culture of** ***B. subtilis*** **LBBMA RI4914.** Maltodextrin (100 g L^-1^) was added as a drying adjuvant.

Maltodextrin at a concentration of 100 g L^-1^ was not sufficient to obtain a solid material with the desired characteristics. In this treatment, it was obtained a sticking yellowish powder with high hygroscopicity (Figure 1), unsuitable, therefore, for storage and handling.

**Figure 2:**
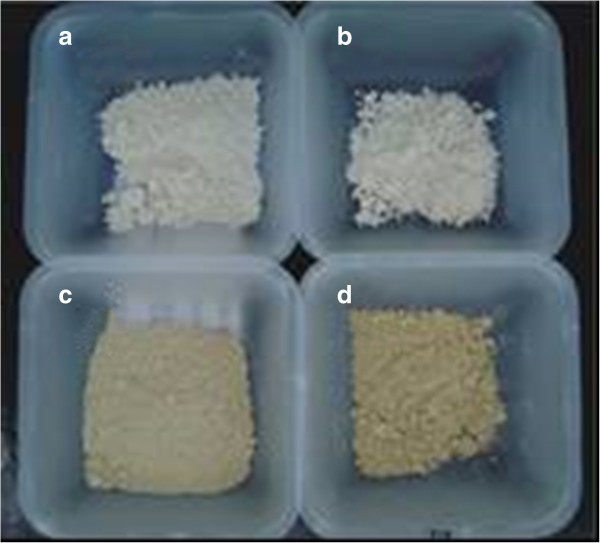
**Solids obtained after spray drying autoclaved culture of** ***B. subtilis*** **LBBMA RI4914 containing the drying adjuvants: a) 250 g L** ^**-1**^ **maltodextrin 10DE; b) 250 g L** ^**-1**^ **maltodextrin 20DE; c) 100 g L** ^**-1**^ **kaolinite; d) 200 g L** ^**-1**^ **kaolinite.**

The addition of maltodextrin 10DE or 20DE at 250 g L^-1^ resulted in a dry solid product with desirable characteristics for storage, handling and transportation (Figure 2a and b). A white, or almost white, slightly sticky and low hygroscopic powder was obtained. The tackiness is similar to pure maltodextrin. There was no effect of different grades of dextrose on the dried product, and the choice between maltodextrin 10DE and 20DE should be made according to the use of the product and the relative cost of each adjuvant.

Both clay concentrations tested (100 or 200 g L^-1^) were effective in obtaining a dried solid product (Figure 2c and d). The material was characterized as a yellowish powder with low hygroscopicity and no stickiness, similar to original kaolinite. The lower concentration (100 g L ^-1^) was considered as the best choice, due to the lower cost with the adjuvant and with storage, transportation and final application.

The solids obtained by spray drying were easily re-suspended in deionized water at room temperature. Maltodextrin was completely solubilized and the clay was put in suspension by simple shaking.

**Figure 3:**
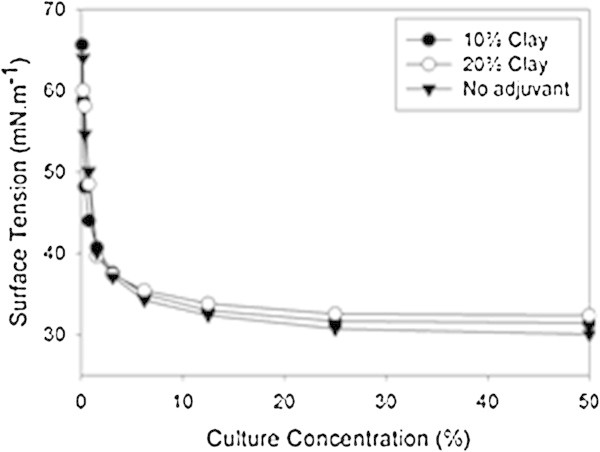
**Effect of clay (100 or 200 g L** ^**-1**^ **kaolinite) on surface tension x dilution curves of crude biosurfactant produced by** ***B. subtilis*** **LBBMA RI4914.**

The addition of drying adjuvants to the crude extract of biosurfactant produced by *B. subtilis* LBBMA RI4914 caused a small change in the surface tension of the surfactant solution. The surface tension of the diluted crude extract in deionized water (1:1) was 30.0 mN m^-1^. This value increased to 31.4 and 32.3 mN m^-1^ when clay was added at concentrations of 100 and 200 g L^-1^, respectively (Figure 3). The data reflect a possible interference of clay with the surface activity of biosurfactants, since the graphics of CMD do not indicate loss of biosurfactant by adsorption to the clay lamellae. This conclusion comes from the observation that, as the samples were diluted, less difference between the values of surface tension of the samples with clay and the crude surfactant control was found.

**Figure 4:**
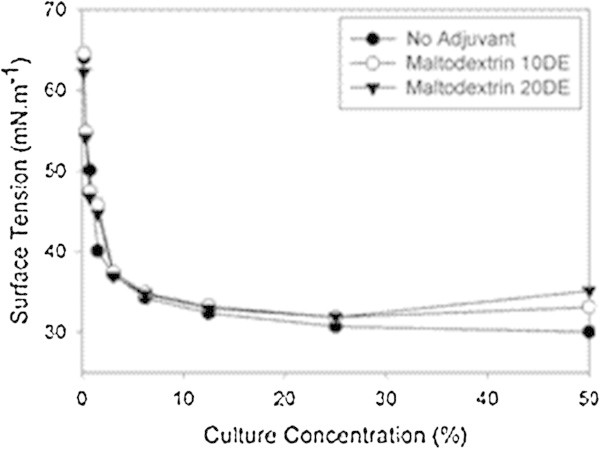
**Effect of maltodextrin 10DE or 20DE (250 g L** ^**-1**^ **) on surface tension x dilution curves of crude biosurfactant produced by** ***B. subtilis*** **LBBMA RI4914.**

**Figure 5:**
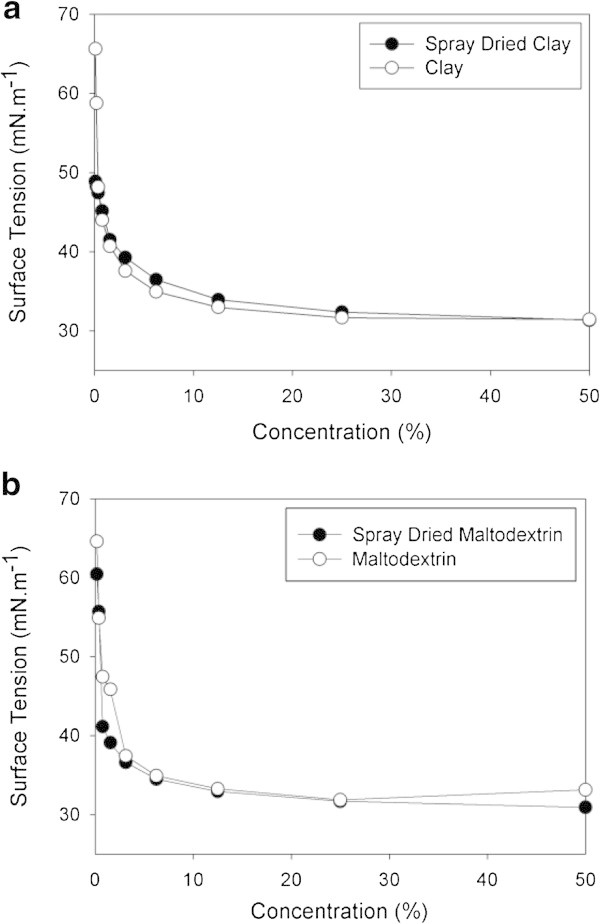
**Effect of drying adjuvants on surface tension x dilution curves of crude biosurfactant produced by** ***B. subtilis*** **LBBMA RI4914, before and after spray-drying: a) kaolinite 100 g L** ^**-1**^ **; b) maltodextrin 10DE 250 g L** ^**-1**^ **.**

At the higher concentration of biosurfactants (and consequently of drying adjuvants), maltodextrin had a higher effect on the surface tension of the crude extract of biosurfactant than clay (compare the surface tensions of the dried formulations and their respective non-dried controls at 50% (1:1) dilution in Figures 4 and 5). Surface tensions of the diluted extract (1:1) were 33.1 and 35.1 mN m^-1^ in the presence of maltodextrin 20DE and 10DE, respectively (Figure 4). These results indicate that different concentrations of dextrose in maltodextrin 20DE and 10DE may be the responsible for the differences between the surface tensions measured in the treatments at the lower (50%) dilution. As the samples were diluted (and consequently the concentration of the drying adjuvant), the difference between the surface tension of the maltodextrin-containing treatments and the non-dried control (no drying adjuvant added) were reduced, similar to the results obtained with clay.

### Surface activity of dried products

The biosurfactant produced by *B. subtilis* LBBMA RI4914 retained its surface activity after dehydration by spray drying and reconstitution (Figure 5). As already reported for other biosurfactants (Darvishi et al. 2011;Desai & Banat 1997;Pornsunthorntawee et al. 2008), the surfactant produced by *B. subtilis* RI4914 was resistant to high temperature, maintaining its stability after autoclaving. However, this is the first report of maintenance of surface activity of a biosurfactant after exposure to a temperature of 170°C, even for a short period. This thermal stability is essential for biosurfactant concentration and recovery by spray-drying, and also reinforces the potential of these molecules for use in biotechnological processes in which thermal stability is necessary, such as in Microbial Enhanced Oil Recovery (MEOR) of high temperature oil reservoirs.

When clay was used as drying adjuvant, there was a subtle difference between the curves of CMD of the crude biosurfactant extracts before and after its dehydration by spray drying. It was observed a faster rise of the surface tension along the dilution, after the clay-crude biosurfactant mixture was spray dried (Figure 5a). This behavior of the CMD curve indicates a lower concentration of biosurfactant in the extract subjected to spray drying, which was attributed to adsorption of molecules to the clay or thermal loss of some molecules. Under conditions of neutral pH, such as used in this work, anionic molecules, such as surfactin, present net negative charge (Shen et al. 2011), which may contribute to their adsorption onto clay in the presence of divalent Mg^2+^ present in the growth medium.

No difference of surface tension was observed between surfactin solutions containing maltodextrin before and after spray drying (Figure 5b), indicating that most of the biosurfactant molecules contained in the initial crude extract were active upon spray-drying and that these molecules were released into solution upon dissolving the dried solid product.

The different results when using clay or maltodextrin as drying adjuvant are attributed to the mechanism of action of these adjuvants during spray drying. The protective mechanism of the compound of interest by maltodextrin, during and after the drying process, is microencapsulation, which consists of forming a wall around the microdroplet of the product (Krishnaiah et al. 2012). The mechanism of action of the clay involves the adsorption of the biosurfactant into its lamellae. However, further studies must be conducted in order to clarify the types of interaction between the biosurfactant molecules and the surface of such adjuvant, as well as what are the factors that interfere with these interactions.

### Stability of dried biosurfactant

**Figure 6:**
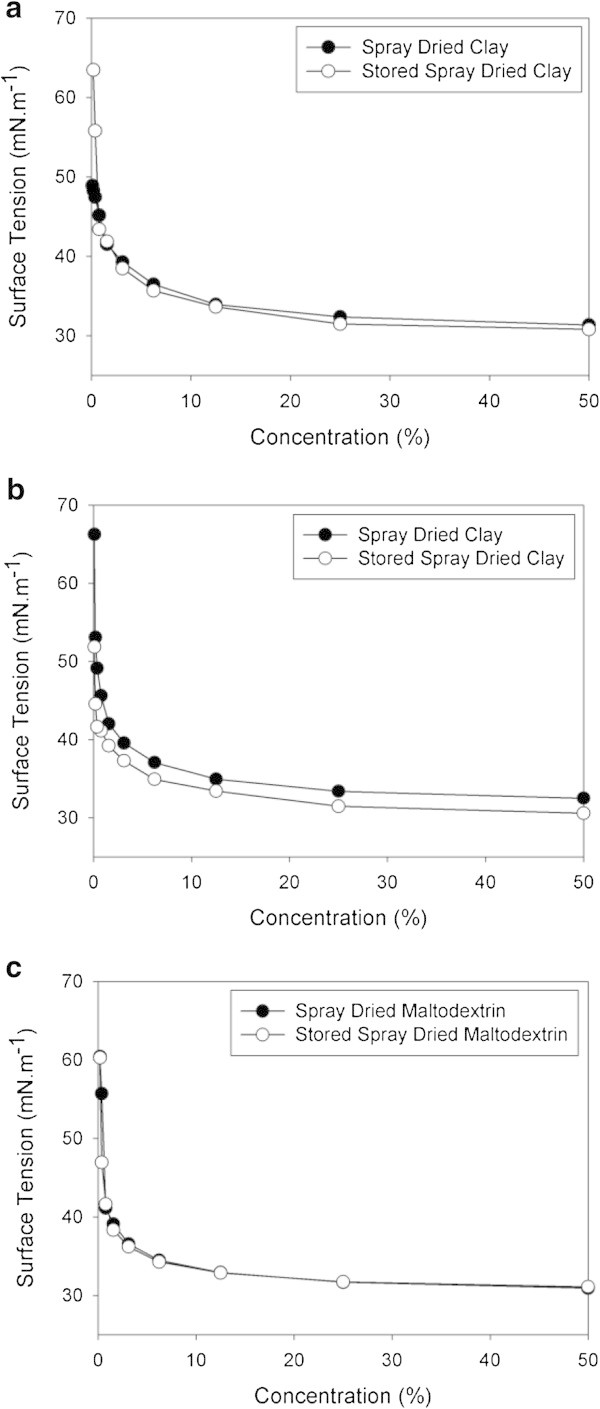
**Surface tension x dilution curves of spray dried crude biosurfactant produced by** ***B. subtilis*** **LBBMA RI4914, one day after spray-drying and after 4 months of storage at environment conditions. a)** kaolinite 100 g L^-1^; **b)** kaolinite 20 g L^-1^; **c)** maltodextrin 10DE 250 g L^-1^.

The surface-active properties of biosurfactant remained unchanged after four months of storage at room temperature and humidity (Figure 6). There was a slight reduction of the ST in the curves of CMD of the clay-based product after storage, this reduction being proportional to the amount of clay used (Figure 6a and b). These data reinforce the hypothesis of adsorption of the biosurfactant to the lamellae of the clay during drying (and not a thermal loss of some molecules), and suggest a possible gradual release of these molecules during storage. This type of interaction, apparently more stable than the interaction between biosurfactant and maltodextrin, may explain the reduction of the concentration of biosurfactant after resuspension of the product containing clay (Figure 5a).

The gradual release of the molecules of biosurfactant from clay can be advantageous, depending on the process wherein the compound is applied (for example, in bioremediation of hydrophobic organic contaminants in soils and sediments). In this case, the clay containing biosurfactant could be incorporated into the soil, to provide a gradual and continuous release of the surfactant molecules in the soil solution. Given the high biodegradability of biosurfactants (Lima et al. 2010), this gradual release can ensure the bioavailability of hydrophobic contaminants for a longer period, thereby facilitating their biodegradation.

In the treatment with maltodextrin, no difference was observed in CMD curves after storage, indicating that all biosurfactant molecules present in the solid were active after four months (Figure 6c). By providing protection of target molecules through microencapsulation, maltodextrin prevents loss of the encapsulated material by avoiding its exposure to adverse conditions, such as those found during the spray drying process and storage (Risch & Reuneccius 1993;Shahidi & Han 1993).

## Conclusions

The spray drying technique was proven effective in the recovery and concentration of surfactin, while maintaining the tensoactive properties of the molecule. The use of drying adjuvants was necessary to obtain a solid product with adequate hygroscopicity and moisture. These compounds do not interfere with tensoactive properties of the surfactant molecules.

Spray drying of biosurfactants open a new horizon in environmental biotechnology, since it leads to a significant reduction of volume and increased stability of the product, thus facilitating its storage and transportation.
